# Subgroup Analysis of Trials Is Rarely Easy (SATIRE): a study protocol for a systematic review to characterize the analysis, reporting, and claim of subgroup effects in randomized trials

**DOI:** 10.1186/1745-6215-10-101

**Published:** 2009-11-09

**Authors:** Xin Sun, Matthias Briel, Jason W Busse, Elie A Akl, John J You, Filip Mejza, Malgorzata Bala, Natalia Diaz-Granados, Dirk Bassler, Dominik Mertz, Sadeesh K Srinathan, Per Olav Vandvik, German Malaga, Mohamed Alshurafa, Philipp Dahm, Pablo Alonso-Coello, Diane M Heels-Ansdell, Neera Bhatnagar, Bradley C Johnston, Li Wang, Stephen D Walter, Douglas G Altman, Gordon H Guyatt

**Affiliations:** 1 Department of Clinical Epidemiology and Biostatistics, McMaster University, Hamilton, Canada; 2 Center for Clinical Epidemiology and Evidence-Based Medicine, West China Hospital, Sichuan University, Chengdu, PR China; 3 Basel Institute for Clinical Epidemiology and Biostatistics, University Hospital Basel, Basel, Switzerland; 4 The Institute for Work & Health, Toronto, Ontario, Canada; 5 Departments of Medicine and Family Medicine, State University of New York at Buffalo, NY, USA; 6 Department of Medicine, McMaster University, Hamilton, Canada; 7 Department of Pulmonary Diseases, Jagiellonian University School of Medicine, Krakow, Poland; 8 Department of Internal Medicine, Jagiellonian University School of Medicine, Krakow, Poland; 9 University Children's Hospital Tuebingen, Department of Neonatology, Tuebingen, Germany; 10 Division of Infectious Diseases & Hospital Epidemiology, University Hospital Basel, Switzerland; 11 Section of Thoracic Surgery, Department of Surgery, University of Manitoba, Winnipeg, Manitoba, Canada; 12 Norwegian Knowledge Centre for the Health Services, Oslo, Norway; 13 Universidad Peruana Cayetano Heredia, Lima, Peru; 14 Department of Urology, University of Florida, College of Medicine, Gainesville, Florida, USA; 15 Iberoamerican Cochrane Center. Hospital de la Santa Creu i Sant Pau, Barcelona, Spain; 16 CIBER de Epidemiología y Salud Pública (CIBERESP), Spain; 17 Health Sciences Library, McMaster University, Hamilton, Canada; 18 Centre for Statistics in Medicine, University of Oxford, Oxford, UK

## Abstract

**Background:**

Subgroup analyses in randomized trials examine whether effects of interventions differ between subgroups of study populations according to characteristics of patients or interventions. However, findings from subgroup analyses may be misleading, potentially resulting in suboptimal clinical and health decision making. Few studies have investigated the reporting and conduct of subgroup analyses and a number of important questions remain unanswered. The objectives of this study are: 1) to describe the reporting of subgroup analyses and claims of subgroup effects in randomized controlled trials, 2) to assess study characteristics associated with reporting of subgroup analyses and with claims of subgroup effects, and 3) to examine the analysis, and interpretation of subgroup effects for each study's primary outcome.

**Methods:**

We will conduct a systematic review of 464 randomized controlled human trials published in 2007 in the 118 Core Clinical Journals defined by the National Library of Medicine. We will randomly select journal articles, stratified in a 1:1 ratio by higher impact versus lower impact journals. According to 2007 ISI total citations, we consider the *New England Journal of Medicine, JAMA, Lancet, Annals of Internal Medicine*, and *BMJ *as higher impact journals. Teams of two reviewers will independently screen full texts of reports for eligibility, and abstract data, using standardized, pilot-tested extraction forms. We will conduct univariable and multivariable logistic regression analyses to examine the association of pre-specified study characteristics with reporting of subgroup analyses and with claims of subgroup effects for the primary and any other outcomes.

**Discussion:**

A clear understanding of subgroup analyses, as currently conducted and reported in published randomized controlled trials, will reveal both strengths and weaknesses of this practice. Our findings will contribute to a set of recommendations to optimize the conduct and reporting of subgroup analyses, and claim and interpretation of subgroup effects in randomized trials.

## Background

The effects of healthcare interventions on the entire study population are of primary interest in clinical trials. It remains appealing, however, for investigators and clinicians to identify differential effects in subgroups based on characteristics of patients or interventions. This analytic approach, termed subgroup analysis, can sometimes be informative - but it is often misleading [1-4].

Investigators frequently conduct subgroup analyses exploring multiple hypotheses [5]. Conducting multiple tests is associated with the risk of false positive results due to the play of chance [3]. This risk is particularly great if subgroup analyses are data driven: that is, when investigators perform numerous *post hoc *subgroup analyses seeking statistical significance. Even when investigators specify a limited number of subgroup analyses *a priori*, the play of chance may still result in identification of spurious subgroup effects.

Sometimes, investigators explore possible subgroup effects by testing the null hypothesis of no treatment effect in each of the relevant subgroups. A claim of subgroup effect is made if a significant effect is observed in one subgroup but not in the other(s) [6,7]. This strategy, however, fails to address the real issue of subgroup analysis: can chance explain the apparent difference between subgroups? This question can be addressed with a formal test of interaction in which the null hypothesis is that the underlying effect across subgroups is the same. In another instance, investigators report and claim the effect of one subgroup of patients while ignore reporting of other subgroups. Investigators may also test the difference of effects between groups according to the study characteristic measured after randomization. The apparent difference of effects may, however, be explained by the treatment intervention itself, or by differing prognostic characteristics in sub-groups that emerge after randomization, rather than by the subgroup characteristic itself. Therefore, this approach to analyzing subgroups is highly problematic [4,8,9].

Many apparent subgroup effects have been proven to be spurious [10]. Misleading subgroup effects can result in withholding efficacious treatment from patients who would benefit, or encourage ineffective or potentially harmful treatments for subgroups who would fare better without. It is, therefore, imperative to critically assess the validity of claimed subgroup effects. One approach is to use seven previously proposed criteria for determining whether apparent differences in subgroup response are likely to be real [11]. These criteria have been widely used to evaluate subgroup analyses in randomized controlled trials (RCTs) and meta-analyses [12-15]. Several new criteria may further facilitate differentiation between spurious and real subgroup effects (Appendix 1).

A limited number of empirical studies have evaluated how trialists conduct and report subgroup analyses, and have revealed several weaknesses (Table 1) [16-21]. Weaknesses include the use of an excessive number of variables and outcomes, inappropriate statistical methods, and insufficient *a priori *specification of variables. A review of subgroup analyses reported in cardiovascular trials [17], for instance, identified one study reported 23 subgroup variables and 17 outcomes. In another review of 27 surgical trials [16], a test of interaction was reported for only 5.8% (3/54) of subgroup hypotheses tested, whereas 72.2% (39/54) claimed subgroup effects. Across six reviews of subgroup analyses, the prevalence of trials claiming at least one subgroup effect ranged from 25% to 60% [16-20]. Two studies - one [18] restricted to trials published in the New England Journal of Medicine, and another [17] restricted to moderate or large sized cardiovascular trials - found that larger sample size was the only study characteristic statistically associated with reporting of subgroup analyses.

**Table 1:** Characteristics of six studies reviewing subgroup analyses in randomized trials

**Study ID**	**Trial area**	**Source of study**	**Number of trials**	**Trial feature for eligibility criteria**
Wang (2007)	Multiple	NEJM (July 2005 to June 2006)	97 (59 reporting subgroup analyses)	No restrictions
Bhandari (2006)	Surgical	Two surgical journals plus NEJM, JAMA, BMJ, and Lancet (Jan 2000 to Apr 2003)	72 (27 reporting subgroup analyses)	No restriction on size and other trial characteristics
Hernandez (2006)	Cardiovascular	Four cardiovascular journals plus "Top Five" (2002 and 2004)	63 (39 reporting subgroup analyses)	Phase 3 parallel trials, n ≥ 100, superiority trials; restricted to main reports
Hernandez (2005)	Traumatic brain	MEDLINE (1966 to Apr 2004), EMBASE (1978 to Apr 2004), CENTRAL (Apr 2004)	18 (11 reporting subgroup analyses)	Phase 3, parallel trials, n ≥ 50 per arm Glasgow Outcome Scale (GOS) at 3 months as outcome
Moreira Jr (2001)	Multiple	NEJM, JAMA, Lancet, American Journal of Public Health (July 1998)	32 (17 reporting subgroup analyses)	No restrictions mentioned.
Assmann (2000)	Multiple	NEJM, JAMA, BMJ, and Lancet (July to Sep 1997)	50 (35 reporting subgroup analyses)	No crossover and cluster trials, n ≥ 50

Despite the merits of these studies, each of them examined only a relatively small number of trials (median 57, range 11-97). None compared the reporting of subgroup analyses in higher impact journals versus other journals; none examined the reporting of subgroup analyses in relation to type of outcomes (e.g. continuous, binary, time-to-event, count, or multinomial); and none specifically examined subgroup analysis reporting for the primary outcome. In addition, none of the previous reviews documented the magnitude of the apparent subgroup effects and magnitude of p-values of interaction tests; none investigated the validity of claimed subgroup effects; none investigated study characteristics associated with claim of subgroup effects; and none addressed the credibility of the claimed subgroup effects.

These shortcomings limit the generalizability of findings and leave important questions unanswered. Therefore, we will conduct a systematic review of RCTs to further inform the current use and reporting of subgroup analyses.

In this study, we have three main objectives. The first is to describe the reporting of subgroup analyses and claim of subgroup effects. The second is to assess study characteristics associated with reporting of subgroup analyses, and study characteristics associated with claim of subgroup effects, both for the primary outcome and for any outcome. The third objective is to examine the analysis and interpretation of subgroup effects conducted for the primary outcome.

## Methods

### Study Design Overview

We will conduct a systematic review of RCTs conducted in humans and published in 2007 in the Core Clinical Journals defined by the National Library of Medicine . To maximize the generalizability of study findings, we will include parallel, cross-over, and factorial randomized trials, and both individual and cluster randomised trials. Unless the authors report findings to the contrary, we will assume no treatment-by-treatment interaction in factorial studies, no treatment-by-sequence interaction in cross-over studies, and no treatment-by-cluster interactions in cluster-randomized studies. We will use the standard methodology for conducting systematic reviews [22].

### Definition of Subgroup, Subgroup Analysis, and Subgroup Effect

For this study, we define a subgroup as a subset of a trial population that is identified on the basis of a patient or intervention characteristic that is either measured at baseline or after randomization.

We define a subgroup analysis as a statistical analysis that explores whether effects of the intervention (i.e. experimental versus control) differ according to status of a subgroup variable. This includes a case in which investigators report a main result and analyze only a subset of patients.

We define a subgroup effect as a difference in the magnitude of a treatment effect across subgroups of a study population. The null hypothesis for a test of a subgroup effect (i.e. subgroup hypothesis) is that there is no difference in the magnitude of a treatment effect across subgroups. We will consider both absolute and relative effect measures in our study.

### Eligibility Criteria

The inclusion criteria are:

1) The study is an RCT;

2) The participants are human;

3) The study is published in 2007 in a core clinical journal (as defined by the National Library of Medicine).

The exclusion criteria are:

1) The report does not include the entire population enrolled in the original study (i.e. the report focuses on a subset of the original study population);

2) The study is explicitly labelled as a phase I trial;

3) The study is exclusively a pharmacokinetic study;

4) The study is reported as a Research Letter.

No restrictions apply with respect to the following aspects:

• Trial design (i.e., parallel, factorial or cross-over);

• Number of trial arms (i.e., two or more);

• Unit of randomization (i.e., individual patient or cluster);

• Type of outcome (i.e., continuous, binary, time-to-event, count, or multinomial);

• Type of trial (i.e., superiority, non-inferiority or equivalence trial);

• Type of report (i.e., main report, longer follow-up report, or interim report);

• Subgroup variables measured at baseline versus after randomization.

• Sample size, length of follow up, and loss to follow up;

• Statistical significance versus non-significance of overall main effects;

### Literature Search

We will search for RCTs published in the Core Clinical Journals in 2007. This group of journals is defined by the National Library of Medicine, includes a total of 118 journals covering all specialities of clinical medicine and public health sciences, and is known as the *Abridged Index Medicus*. We will run the Medline search using the OVID platform and a search strategy (Appendix 2) developed with the help of an experienced librarian.

### Random Sampling of Citations

We will stratify the Core Clinical Journals into higher and lower impact journals. For this study we define higher impact journals as the five journals with the highest total citations in 2007: the *New England Journal of Medicine, JAMA, Lancet, Annals of Internal Medicine*, and *BMJ*. Lower impact journals consist of the remaining Core Clinical Journals. We will randomly sample the journal articles, with 1:1 stratification by journal type (i.e. higher and lower impact). We will continue the random sampling process until the number of eligible studies meets our required sample size.

### Review process

Teams of two trained reviewers will perform citation and full text screening and data abstraction, in duplicate and independently, including the selection of the primary outcome (using pre-specified criteria - see below), selection of the pair-wise comparison for analysis (if there are three or more arms). Each team will attempt to resolve discrepancies by consensus or, if discrepancy remains, through discussion with one of two arbitrators (XS, GHG). The arbitrator will independently review the trial report before discussing it with the reviewers. Before the review formally starts, we will conduct calibration exercises to ensure consistency across reviewers. We will use electronic forms, developed with Microsoft Access and Excel, for study screening and data extraction. The forms will be standardized and pilot-tested, and detailed written instructions will be developed to assist with study screening and data extraction.

### Study Screening

Two reviewers will independently screen the title and abstract of each randomly chosen citation for potential eligibility. In the title and abstract screening, they will judge only if the study is a randomized controlled trial enrolling human participants. Two reviewers will then independently screen the full text of the potentially eligible trials to determine eligibility.

At the full text screening stage, the reviewers will select a primary outcome for eligible studies, using the following strategy: If the report specifies a primary outcome, we will select it as the primary outcome; if the report specifies more than one primary outcome (i.e. co-primary outcomes), we will select the one with the largest number of subgroup analyses; if outcomes have the same number of subgroup analyses, we will select the one with the greatest relevance to patients according to a pre-defined outcome hierarchy, and if more than one outcome are in the same category, we will take the first reported outcome in the abstract (Appendix 3). If the report does not specify a primary outcome, we will select the outcome used for the study sample size calculation, but if there is no sample size calculation reported or if there is a sample size calculation for several outcomes, we will proceed as detailed in the previous sentence.

Reviewers will also identify a pair-wise comparison of interest, using the following strategy. If there are only two groups, we will use them for the pair-wise comparison. If there are three or more groups, we will select the comparison that was clearly and explicitly defined as the primary comparison in the study report; if the primary comparison was not explicitly defined, we will select the comparison that reports the largest number of subgroup analyses for the selected primary outcome; if more than one comparison reported the same largest number of subgroup analyses, we will select the comparison that reports the smallest interaction p value; if the interaction p value is not available, we will select the one that has the smallest p value for the main effect.

### Data Abstraction

#### Study Characteristics

We will extract information on funding sources, clinical area, type of intervention, trial design (parallel, cross-over, or factorial), trial type (superiority, non-inferiority, or equivalence), unit of randomization (randomization at individual or cluster level), methodological characteristics of trials (allocation concealment; blinding of patients, healthcare givers, data collectors, outcome adjudicators, or data analysts; stopping trials early for benefit), number of participants randomized for the selected comparison, and total number of participants randomized.

We will categorise the selected primary outcome, according to whether it is a composite endpoint, whether the results are statistically significant, and the type of outcome variable (time-to-event, binary, continuous, count, or multinomial). We will record the type of effect measure for the selected primary outcome. If more than one effect measure is used for binary, time-to-event, or count outcomes, we will use a hierarchical approach to select an effect measure, as follows:

• Select the effect measure that the investigators clearly indicated as the effect measure for the primary analysis;

• Select the effect measure on which the subgroup analysis is reported and a subgroup effect is claimed;

• Select the measure that yields the smallest reported p-value of the main effect;

• Otherwise, use the following order for binary outcomes: risk ratio > odds ratio > relative risk reduction > risk difference; and the following for time-to-event outcomes: hazard ratio > incidence rate ratio > ratio of cumulative incidence > ratio of time > difference in incidence rate > difference in cumulative incidence > difference in time

If no effect measure is reported but data for a 2 × 2 table are available for the primary outcome, we will calculate risk ratios.

For binary, time-to-event, and count primary outcomes, we will document their point estimates and 95% confidence intervals for the main effects, as well as - whenever possible - events and number of patients in a 2 × 2 table. For continuous outcomes, we will document the number of patients analyzed in the experimental and control groups, and the summary measure (i.e. means, medians) and associated measure of precision (i.e. inter-quartile range, 95% confidence interval, standard deviation, or standard error). We will not document the magnitude of the main effect for multinomial primary outcomes.

#### Reporting of subgroup analyses

We will record whether trials report subgroup analyses for any outcomes (i.e. primary or secondary), the number of outcomes for which subgroup analyses are reported, the type of outcomes, the number of subgroup variables reported in the trial report, the number of subgroup analyses that were most likely conducted, the number of subgroup analyses reported, whether any subgroup analysis was specified a priori, and whether any subgroup effect was stated to have been analyzed by a test of interaction. We will also document the above information specifically for the primary outcome.

We will consider a subgroup analysis has been reported if: 1) the investigators report a point estimate and an associated confidence interval or a p-value for one or more subgroups of the study original population, 2) the investigators report the magnitude of difference in the effect according to status of a subgroup variable, 3) the investigators report results from an interaction test, or 4) the investigators explicitly state that they conducted subgroup analyses but do not report any of the data mentioned above.

#### Claim of subgroup effects

We will record whether trials claim a subgroup effect for any outcomes (i.e. primary or secondary outcome), number of subgroup effects claimed in the trial report, and type of outcomes used for the claim. We will judge the strength of the claim based on the inferences drawn by the investigators in the abstract or discussion section. We will also document the above information specifically for the primary outcome.

We will consider a subgroup effect is claimed if, in the abstract or discussion of the trial report, the investigators state that the effects of intervention differed, or may have differed, according to status of a subgroup variable.

We will classify the strength of a claim according to four categories, and have defined these categories as below:

1) Strong claim of a definitive effect: The authors convey a conviction that the subgroup effect truly exists.

2) Claim of a likely effect: The authors convey a belief that the subgroup effect likely exists.

3) Suggestion of a possible effect: The authors suggest a subgroup effect and convey an uncertainty whether the subgroup effect exists.

4) No claim of a subgroup effect: The authors do not make a claim of a subgroup effect.

We have developed explicit criteria to judge the strength of claim (Table 2).

**Table 2:** Criteria for judging the strength of a subgroup claim

**Criteria**	**Strong claim**	**Claim of a likely effect**	**Suggestion of a possible effect**
1. Did the investigators claim the effect in the abstract?	Yes	Possible	No

2. Did the investigators claim the effect in the conclusion of abstract?	Possible*	No	No

3. Did the investigators claim the effect in the discussion?	Yes	Possible	Yes

4. Did the investigators use the descriptive words (e.g. appear/seem to be, may, and might) to soften their statements of the claims?	No	Possible	Possible

5. Did the investigators used descriptive words (e.g. particular, and special) to strengthen the statement of the claims	Possible	No	No

6. Were the authors obviously cautious about the apparent subgroup effect? (e.g. they stated the subgroup effect did not meet some of important criteria to believe a subgroup effect)	No	Some caution possible	Yes

7. Did the investigators indicate the apparent effects need to be explored in the future studies (i.e. hypothesis generating)?	No	Possible say desirable to confirm	Yes

* If a claim appears in the conclusion section of the abstract, it is considered a strong claim.

#### Analysis of subgroup effect for the primary outcome

We will document, for each subgroup analysis, whether the subgroup variable is a baseline characteristic or based on an after-randomization event, whether the investigators specified the variable *a priori*, whether the investigators specified the direction *a priori*, whether the subgroup variable was used as a stratification factor in randomization, the type of tests used for analyzing subgroup effects (test of significance of individual groups, interaction test, or both), the statistical approaches used for a test of interaction, and the methods of adjusting for multiple interaction effects.

We will also document, whenever possible, the 2 × 2 data, the reported point estimate, 95% confidence interval, and p-value of the effect of each subgroup, as well as the reported p-value of the interaction test.

#### Interpretation of claimed subgroup effect for the primary outcome

For each of the claimed subgroup effects, we will further document whether the authors provided a supportive biological rationale or cited external evidence that is consistent with the observed subgroup effect, whether the authors indicated that the pre-specified direction was correct, or that they indicated the observed subgroup effect was consistent across closely related outcomes.

### Sample Size

We conducted a pilot study including 139 randomized trials. The results showed that 62 (44.6%) trials reported subgroup analyses for any outcome, and 41 (29.5%) reported for the primary outcome; 27 (19.4%) trials claimed subgroup effect for any outcome, and 18 (12.9%) claimed for the primary outcome.

We calculate the sample size based on the examination of study characteristics associated with claim of subgroup effects for any outcome. In our regression of study characteristics with claim of subgroup effects, we will include 6 study characteristics, a total of 9 categories of variables. We will require 10 events (i.e. claim of subgroup effect) per category to examine the association, resulting in a total of 90 events (and at least 90 total non-events). Given the results of pilot study, we will require a total of 464 trials for this study.

### Statistical Analysis

We will assess agreement between reviewers for study inclusion at the full text screening stage, reviewers' judgments whether the investigators reported a subgroup analysis, claimed a subgroup effect, pre-specified the subgroup hypothesis, or used the interaction test. We will calculate both crude agreement and chance-corrected agreement. We will interpret the agreement statistics using the guidelines proposed by Landis and Koch [23]: kappa values of 0 to 0.20 represent slight agreement, 0.21 to 0.40 fair agreement, 0.41 to 0.60 moderate agreement, 0.61 to 0.80 substantial agreement, and greater than 0.80 almost perfect agreement.

We will calculate the proportions of trials reporting at least one subgroup analysis for the primary outcome and for any outcome. Treating the reporting of a subgroup analysis as the dependent variable, we will conduct univariable and multivariable logistic regression analyses to examine its association with the pre-specified study characteristics for both the primary outcome and for any outcome.

We will also calculate the proportions of trials claiming a subgroup effect for the primary outcome and for any outcome in trials that report a subgroup analysis, and conduct univariable and multivariable logistic regression analyses to examine the association of pre-specified study characteristics with claim of a subgroup effect for the primary outcome and for any outcome.

Our pre-specified study characteristics for the regression analyses are: average sample size per study arm, journal type (high vs. lower impact journals), source of funding (partially or completely funded by private for profit organization vs. others), statistical significance of the main effect, trial area (medical vs. surgical), number of pre-specified primary outcomes (used for the regression of reporting of subgroup analyses only), number of subgroup analyses (used for the regression of claim of subgroup effects only). We hypothesize that trials are more likely to report subgroup analyses or claim subgroup effect if they have larger sample size, are published in higher impact journals, receive funding from for profit organizations, do not achieve statistical significance for the main effect, investigate medical versus surgical interventions, have more pre-specified primary outcomes, and larger number of subgroup analyses. In the multiple logistic regression analysis for reporting of subgroup analysis, we will also examine the interaction of source of funding and significance of main effect.

We will describe the details of reporting of subgroup analyses and claim of subgroup effects for both any outcome and specifically for the primary outcome. If a variable, in both univariable and multivariable analyses, is found to be significantly associated with reporting of a subgroup analysis and/or claim of a subgroup effect, we will also present the above information stratified by the type of journal.

We will describe the details of analysis of subgroup effects for the primary outcome by journal type (i.e. five highest impact journals versus other journals), and by claim versus no claim of a subgroup effect. We will also describe the details of interpretation of claimed subgroup effects by journal type.

## Discussion

Our study is designed to comprehensively address the analysis, reporting, and claim of subgroup effects in a representative sample of recent RCTs. This study protocol follows the publications of two other protocols [24,25] which reflects our continuing efforts to make objectives and design of methodological studies more transparent.

### Strengths and limitations

Our study has several strengths. First, we will employ rigorous systematic review methods including explicit and reproducible eligibility criteria, sensitive search strategies, and the use of standardized, pilot-tested forms accompanied by written instructions for study screening and data extraction. Teams of two trained reviewers will independently and in duplicate conduct study screening. We will also undertake calibration exercises and pilot data extraction to enhance consistency between reviewers before embarking on data abstraction. Second, our eligibility criteria are broad, and compared to the previous empirical studies our study findings will be more generalizable. Third, we conducted a pilot study to calculate the required sample size for the definitive study. Finally, our study will be the largest empirical study of subgroup analyses which will allow us to reliably address a number of important questions that have not been addressed by existing reviews.

Our study also has several limitations. It will be based on reported trial information, and our findings may be vulnerable to underreporting or selective reporting [26]. The limited space allowed by medical journals for reporting on trials may prevent authors from sufficiently reporting relevant information on subgroup analyses. Consequently, the proportion of trials reporting subgroup analyses is probably smaller than the proportion of trials actually conducting subgroup analyses, and the number of subgroup analyses reported in each trial is probably smaller than the actual number of conducted subgroup analyses. In relation to this problem, we will also estimate the number of subgroup analyses that were most likely conducted. Similarly, other details about subgroup analyses, such as *a priori *specification of the subgroup hypothesis and direction, may also be under-reported.

Our study does not include all medical journals, and our findings may not be applicable to journals outside our sample. Our study, however, includes many more journals than the previous studies that typically included high impact journals or specialty journals only. We chose the Core Clinical Journals because they cover all clinical and public health areas, and include all major medical journals. We consider that the quality of studies in these journals will be no worse than that in other journals, and expect that the quality of subgroup analyses reported in other journals will be no better than that in the Core Clinical Journals.

Our study will involve reviewers' judgement of the strength of the claim of subgroup effect, and the determination of strength may be subjective and vary across reviewers. We have developed detailed written instructions to assist reviewers in judging the strength, and will check the inter-reviewer agreement.

### Implications of this study

Although a few empirical studies restricted to certain disease areas or journal type have found a significant association between sample size and reporting of subgroup analyses, factors that drive reporting and claiming of subgroup effects in a more representative set of trials remain uncertain. The results of this study will provide robust, generalizable, and reliable evidence on the factors that impact reporting and claiming of subgroup effects.

Considerable work, including methodological advocacy [3,27-31] and empirical investigation [5,18,19], has been done to inform the conduct of subgroup analyses. However, few reports have systematically developed the framework of analysis, reporting, claim, and interpretation of subgroup effects. The findings of this study will further aid in the development of recommendations for adequate reporting, and appropriate analysis, claim, and interpretation of subgroup effects.

Claimed subgroup effects are of primary interest to clinicians, investigators and other users. Claims of spurious subgroup effects can distort clinical practice and public health decision making, with serious consequences for patients and unnecessary expenditures. Methodological safeguards have been proposed to protect from spurious subgroup findings [4,10,30], but empirical evidence of their validity is limited. The results of this study will reveal the extent to which the investigators considered methodological safeguards in their claims, and provide some evidence regarding the extent to which claims of subgroup effects are valid.

The findings of the SATIRE study may influence recommendations on reporting, conduct, claim, and interpretation of subgroup analyses. These will be of particular interest to the stakeholders that have direct influence on trial design, analysis, and reporting, including investigators, health decision makers, guideline developers, funding agencies, and medical journal editors.

## Competing interests

The authors declare that they have no competing interests.

## Authors' contributions

XS and GHG conceptualized the study. All authors contributed to design of the study and read and approved the manuscript. XS developed the first draft of the manuscript and incorporated comments from authors for successive drafts.

## Appendices

### Appendix 1. The eleven criteria for assessing credibility of claimed subgroup effects

• *Is the subgroup variable a characteristic at randomization?*

• Is the effect suggested by comparisons within rather than between studies?

• Does interaction test suggests a low likelihood that chance explains the apparent subgroup effect?

• *Is the significant interaction effect independent of other potential subgroup effects?*

• Was the hypothesis specified a priori?

• *Was the correct direction of subgroup effect specified a priori?*

• Was the subgroup effect one of a small number of hypothesized effects tested?

• Is the magnitude of the subgroup effect large?

• Is the interaction consistent across studies?

• *Is the interaction consistent across closed related outcomes within the study?*

• Is there indirect evidence that supports the hypothesized interaction?

*The new criteria are italicized*.

### Appendix 2: Search strategy

1. exp Randomized Controlled Trials/

2. (randomized controlled trial$ or randomised controlled trial$).mp. [mp = title, original title, abstract, name of substance word, subject heading word]

3. (randomized trial$ or randomised trial$).mp. [mp = title, original title, abstract, name of substance word, subject heading word]

4. (randomized clinical trial$ or randomised clinical trial$).mp. [mp = title, original title, abstract, name of substance word, subject heading word]

5. 1 or 2 or 3 or 4

6. limit 5 to (English language and humans and "core clinical journals (aim)" and yr="2007")

### Appendix 3: Hierarchy of outcomes

I. Mortality

1) all cause mortality

2) disease specific mortality

II. Morbidity

1) cardiovascular major morbid events

2) other major morbid events (e.g. loss of vision, seizures, fracture, revascularization)

3) recurrence/relapse/remission of cancer/disease free survival

4) renal failure requiring dialysis

5) hospitalizations

6) infections

7) dermatological/rheumatologic disorders

III. Symptoms/Quality of life/Functional status (e.g. failure to become pregnant, successful nursing/breastfeeding, depression)

IV. Surrogate outcomes (e.g. viral load, physical activity, post operative atrial fibrillation)
